# Dental caries and actual utilization of dental services among primary school children in Egypt: a cross-sectional study

**DOI:** 10.1186/s12903-025-06414-3

**Published:** 2025-07-15

**Authors:** Mariam Osman Mohamed, Basman Elsayed Hamza, Rabaa Mahmoud Aboubakr, Nasr Mohamed Attia

**Affiliations:** 1 Department of Public Health and Preventive Dentistry, Faculty of Dentistry, Mansoura University, Mansoura, Egypt; 2 Public Health Department and Preventive Dentistry, Arab Republic of Egypt, Mansoura University, Al- Daqhalia Governorate, Mansoura, Egypt

**Keywords:** Children, Dental caries, Dental services, Parental fear, Utilization

## Abstract

**Background:**

Understanding factors influencing dental service utilization and dental caries assists dental professionals in addressing challenges within their field.

**Objectives:**

Assess the prevalence of dental caries, the actual pattern of dental service utilization, and factors affecting them, such as parents’ 1dental fear, demographic, and socio-demographic variables among primary school children.

**Methods:**

This cross-sectional study included 1075 participants aged 6 to 12 years old, recruited from primary schools in Mansoura city, Egypt. This study was carried out in two stages; first, a well-structured questionnaire was used to collect data on socio-demographic variables and parental fear. Second, clinical examination was performed to measure dental caries according to the World Health Organization (WHO) diagnostic criteria and to record the dental services utilized. A multi-stage cluster random sampling technique was used to select the study sample. Data was analyzed statistically using regression analysis, Chi-square, and Fisher’s exact tests.

**Results:**

The prevalence of dental caries was higher in primary teeth than in permanent teeth (59.3% vs. 35.8%). Likewise, a higher percentage of children had fillings in their primary dentition (14.1%) than in their permanent dentition (5.1%). 6–8 yearss age, of 6–8 years, significantly impacted dental caries prevalence and service utilization. Being in private schools significantly reduced dental caries in both types of dentitions (DMFT: Regression Coefficient (B) =-0.160, P-value (P) = 0.032), (deft: B=-0.459, *P* = 0.007). Children whose fathers had a higher educational level demonstrated lower dental caries scores in both dentitions (DMFT: B=-0.300, *P* = 0.023), (deft: B= -0.429, *P* = 0.035). A higher utilization rate of dental services was noted among children from families with higher incomes (Odds Ratio (OR) = 2.607, *P* = 0.006). Parental fear was significantly correlated to an increased dental caries prevalence and reduced preventive services (*P* ≤ 0.05).

**Conclusion:**

Despite the relatively high prevalence of dental caries among study participants, the utilization of dental services was low. Age, number of children, and parental-related factors were predictors of caries prevalence and dental service utilization. This underscores the importance of implementing nationwide educational initiatives aimed at schoolchildren’s parents to improve their understanding of accessible dental care options.

**Clinical trial number:**

Not applicable.

**Supplementary Information:**

The online version contains supplementary material available at 10.1186/s12903-025-06414-3.

## Introduction

Dental treatment for young children is usually provided only with parental consent. Parents are the main decision-makers on matters affecting child health. Parents’ cultural beliefs, awareness about preventive dental visits, the care of primary teeth, and concern for oral health affect children’s oral health habits [1, 2].

The use of health services reflects how well the healthcare system works, and this should encourage equitable access to healthcare regardless of a population’s socioeconomic or social background or health in general. There are still disparities in the use of dental services in many nations [3]. Dental appointments are crucial to the use of healthcare services. Positive dental experiences allow kids and their caregivers or parents excellent opportunities to apply preventive measures and receive information about oral hygiene [4].

Different familial factors affect the utilization of dental services, such as parents’ educational level, socioeconomic status, beliefs, and perceptions about their children’s dental health conditions. Age, gender, dental insurance, and dental fear affected oral health service utilization [5, 6].

Few studies reported the utilization rate of different restorations and preventive regimens among schoolchildren. A study in Saudi Arabia (2021) showed a 26.1% utilization rate of pit and fissure sealants versus 22.8% stainless steel crowns [7]. Another study in Brasilia (2022) showed that most participating parents with low educational levels preferred nonconservative dental treatments for their children, while parents with a higher educational level preferred conservative intervention [8]. In Egypt, a study reported that 25% of primary school children had dental fear; about half of them had severe dental fear. Additionally, they found that dental fear has a direct relationship with decayed permanent teeth and an inverse relationship with restored permanent teeth. Furthermore, it was shown that several factors contribute to the limited use of dental services among Egyptian school children, including economic challenges, medical insurance, and parents’ dental fear [9].

Although the issue is significant, few studies in Egypt have explored the interrelationship between dental caries, dental fear, and access to dental services. While several researchers have examined the impact of children’s dental fear on pediatric dental treatments, limited attention has been given to parental dental fear and its influence on dental service utilization in Egypt. Moreover, only a single study to date has provided a detailed account of the types of dental services utilized by Egyptian children. Therefore, this study aims to fill this critical gap by offering a comprehensive analysis of the factors associated with dental caries and the actual utilization of dental services. The findings aim to inform policymakers about key determinants of dental service use, thereby supporting more effective planning and implementation of oral health initiatives.

## Subjects and methods

### Study design, location, and population

This cross-sectional study was carried out at primary schools in Mansoura City. The study included boys and girls aged 6 to 12, who were not undergoing orthodontic treatment and had no disabilities.

### Ethical consideration

The study proposal was submitted to the Ethics Committee at the Faculty of Dentistry at Mansoura University (0203023). Participation in this study was completely voluntary. Before collecting data, A written informed consent to participate was obtained from the parents or legal guardians of all participants under the age of 16. Furthermore, verbal approval was obtained from the children after explaining the study’s goals and procedures in a manner suitable for their age. They were aware that their participation in the study was optional and that they could leave without facing any consequences.

### Sample size calculation

Sample size was calculated using the online program Open Epi, an open-source epidemiologic statistics for public health (http://openepi.com/SampleSize/SSPropor.htm). For dental service utilization, the sample size was calculated to be 738 based on the total population number (57814), Care index of 37.4%, Confidence level of 99%, Precision level of 5%, and Design effect of 1.2 [10]. 1500 questionnaires were distributed to compensate for non-response.

### Sample selection

57,814 students enrolled in 19 private and 50 public schools in the 2022–2023 school year, in Mansoura. These educational institutions were distributed between the East and West districts, with 28,154 and 29,660 students, respectively. A multi-stage cluster sampling method was used to select participants. Initially, 15 schools were randomly selected using a number generator: 10 from the public sector and 5 from the private sector of both districts. Then, a sample of classes was selected randomly from chosen schools to represent all primary grades, from first to sixth. Finally, a simple random sampling method was used to select 1500 children from the selected classes.

**Fig. 1:**
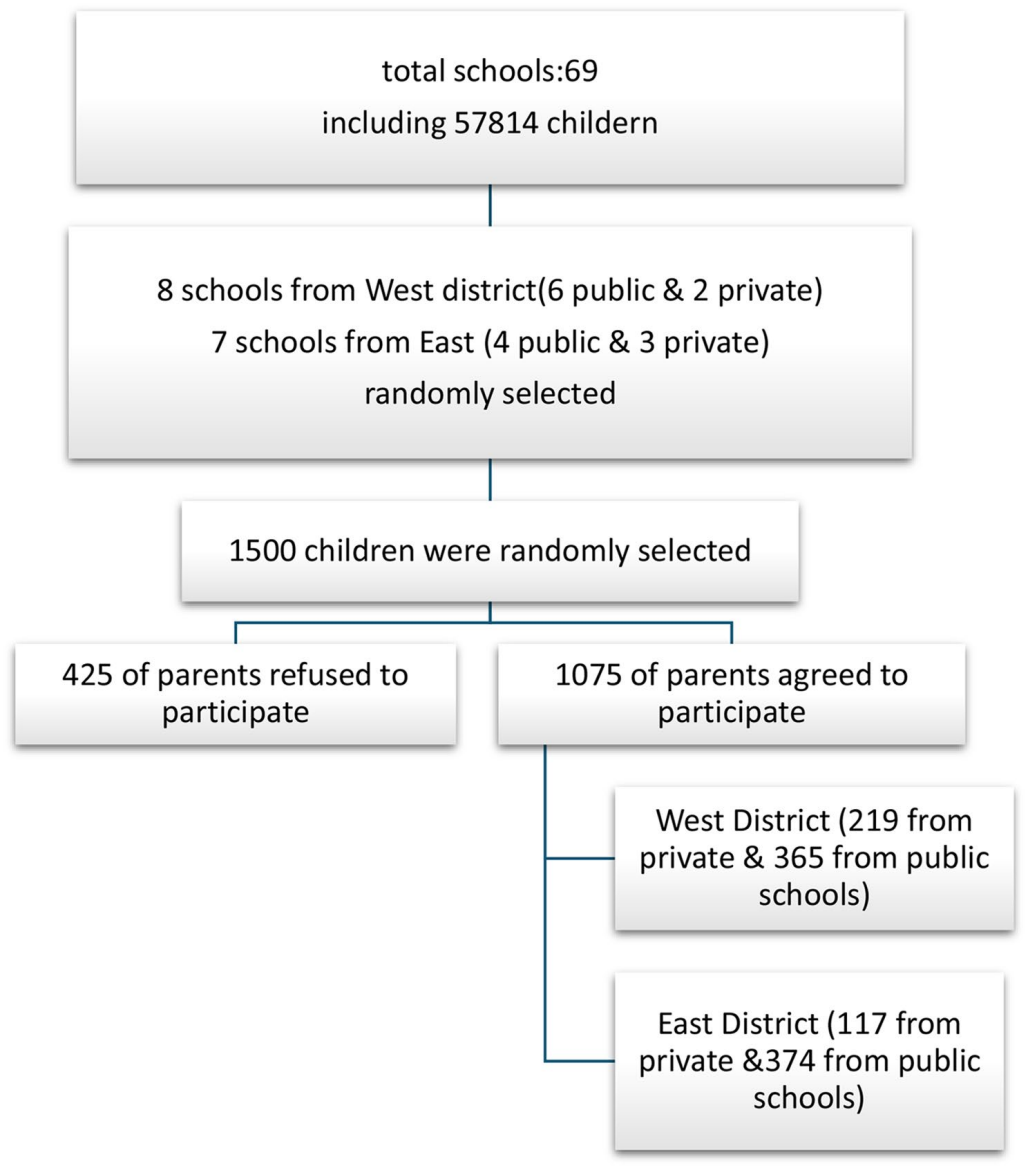
Students’ distribution at primary schools in mansoura city

### Data collection

The survey items were extracted from a published study after obtaining permission [10]. The questionnaire language was Portuguese, which was translated into Arabic by a bilingual expert. Then, a second bilingual expert, who hasn’t seen the original, translated the Arabic version back into Portuguese. The two Portuguese versions (original and back translated) are compared to check for meaning accuracy and consistent and subsequently validated for content validity and reliability. The English version of the questionnaire is provided as Supplementary File (1).

Regarding questionnaire content validity, the item validity index (I-CVI) was good to excellent and ranged from 0.80 to 1. The scale validity index (S-CVI) was assessed to be 1.0 for the parents’ dental fear scale.

Concerning questionnaire reliability, pilot testing was performed utilizing a group of 30 parents who filled out the questionnaire twice, with a minimum interval of one week. They were asked to provide feedback regarding the clarity and comprehensibility of the questions and response options. Cronbach’s alpha coefficient was used to assess the internal consistency of the questionnaire. The initial total Cronbach’s alpha score was 0.658 and interclass correlation coefficient (ICC) scores ranged between 0.1 and 0.194, indicating a need for revision. Based on parents’ feedback, the final version of the questionnaire was modified accordingly. A second pilot study was conducted using the same sample. After the modifications, the parents completed the questionnaire again under the same conditions. As a result, the total Cronbach’s alpha score increased to 0.8, and ICC scores ranged between 0.9 and 1.0, confirming the improved reliability of the final version of the questionnaire.

The questionnaire comprised two sections. Section 1: the demographic and socio-demographic characteristics of the family, including the child’s age, school type, medical history, and number of siblings, as well as parental factors such as age, occupation, education, and household income. Section 2 assessed parents’ dental fear through four questions: (1) How would you feel about tomorrow’s dental appointment? (2) What are your emotions while waiting in the dentist’s reception area? (3) How do you feel while sitting in the dental chair as the dentist prepares the instruments? (4) What do you feel when the dentist works near your gums? The questionnaires were distributed to the parents through school personnel. Study participants assessed their dental fear using a three-point scale (1 = relaxed, 2 = somewhat fearful, and 3 = fearful).

In the results section, fear responses were categorized as either “present” or “absent” based on participants’ answers to standardized fear-related questions. A response indicating any level of fear (e.g., 2 = somewhat fearful or 3 = fearful) was classified as “fear present,” while responses indicating no fear (e.g., 1 = relaxed) were classified as “fear absent.” This binary categorization was applied for clarity in data analysis.

Intra-examiner consistency was examined before the study. 25 children were examined and diagnosed according to World Health Organization (WHO) criteria twice within a week’s interval. The results of the two examinations were compared, and the Kappa agreement value was 90%.

The DMFT and deft indices were used to measure dental caries in permanent and primary teeth, respectively, according to WHO diagnostic criteria [11]. The care index was determined using the equation ( $\:\frac{f}{d+m+f}$) x100, while the restorative index was computed as ( $\:\frac{f}{d+f}$) x100. The unmet treatment need index was calculated using the formula ( $\:\frac{d}{d+f}$) x 100.

Children were screened to record the actual utilization of dental services, including preventive dental services (including pit and fissure sealant, topical fluoride application, and dental scaling), restorative dental services (composite, glass-ionomers, and amalgam), pulp therapy, and stainless-steel crowns. Extractions, space maintainers, dental radiographs, and experienced general anesthesia (GA) for dental treatments were registered. Data on fluoride applications, scaling, pulp therapy, dental radiographs, and general anesthesia were collected using a well-structured parent-completed questionnaire. To assess the accuracy of parental recall, the questionnaire included specific questions about whether the service was provided, as well as the time and place it was received.

### Statistical analysis

IBM SPSS Statistics version 26 was employed to organize, tabulate, and analyze the gathered data. The demographic and socioeconomic profiles of the participants were summarized using descriptive statistics. Chi-square and Fisher’s exact tests were conducted to determine the association between different dental services and parents’ dental fear. Statistical significance was established at a p-value ≤ 0.05. The associations between demographic & socio-demographic variables and dependent variables (caries prevalence and service utilization) were examined by using univariate (unadjusted analysis) and multivariate (adjusted analysis) linear regression and binary logistic regression tests, respectively. Covariates were selected based on a p-value ≤ 0.05 in the model.

## Results

### Participants’ demographic and sociodemographic characteristic

The study included 1,075 children with an equal split between boys (538) and girls (537). The mean age of children was 9.904 ± 1.205 years. Questionnaires were completed by 795 mothers (74%) and 280 fathers (26%). Regarding educational institutions, 336 children (31.4%) were enrolled in private schools, while the remaining 739 (68.7%) attended government schools. Family size varied from one to seven children, with 40% of families having three children and a mere 1.2% having six children. An examination of birth orders showed that 44.8% of children were eldest siblings, 29.2% were second in line, and a mere 0.1% were the youngest in their families. According to health, 4.9% of children had ongoing medical conditions, while 95.1% were illness-free. Maternal education levels varied, with 95 (8.8%) having limited schooling, 283 (26.3%) completing high school, and 697 (64.8%) holding college degrees. Regarding maternal employment, 68.1% were stay-at-home mothers, while 31.7% worked outside the home. As for fathers, 133 (12.4%) did not progress beyond primary education, and 677 (63%) were college-educated. Employment revealed that 951 fathers (88.5%) held professional positions, while only 8 (0.7%) were unemployed. Concerning household finances, 733 (68.2%) families reported their income and their expenditures, whereas 132 (12.3%) families borrowed to meet their needs. A mere 210 (19.5%) families managed to set aside money for savings.

### Distribution of dental caries among study participants regarding their gender and type of school

The study shows that 54.2% of children had dental caries in their primary teeth compared to 34% in their permanent teeth. Similarly, 14.1% of children had fillings in their primary teeth, while only 5.1% had fillings in their permanent teeth. Overall, dental caries prevalence was higher in primary teeth (59.3%) than in permanent teeth (35.8%). The care index (CI) was 12.9% among the study sample, higher in females than males, and higher in private schools than governmental ones. The restorative index (RI) was 13.1%, showing the same gender and school distribution as the CI. The unmet treatment need index (UTNI) was 89.9%, with males scoring higher (88.9%) than females (85.5%), and the score was lower in private schools compared to governmental schools. (Table 1)

**Table 1:** The total number of decayed, missed, and filled teeth and the dental care utilization among studied children according to gender and school type

	Variables	Gender		School type		Total N (%)
		Males	Females	Public	Private	
Permanent teeth	D	153	212	269	96	365 (34%)
	M	3	1	3	1	4 (0.40%)
	F	19	36	32	23	55 (5.1%)
	DMFT	175	249	304	120	424 (35.8%)
	Care index	10.9%	14.5%	10.5%	19.2%	12.9%
	Restorative index	11.0%	14.5%	10.6%	19.3%	13.1%
	Unmet treatment needs index	88.9%	85.5%	89.4%	80.6%	86.9%
Primary teeth	d	314	269	423	160	583 (54.2%)
	e	62	73	110	25	135 (12.6%)
	f	78	74	96	56	152 (14.1%)
	deft	454	416	629	241	870 (59.3%)
	Care index	17.2%	17.8%	15.3%	23.2%	17.5%
	Restorative index	19.9%	21.6%	18.5%	25.9%	20.7%
	Unmet treatment needs index	80.1%	78.4%	81.5%	74.1%	79.3%

The total examined children was 1075

**Table 2:** Comparison of mothers’ and fathers’ reported utilization of dental services for their children

Type of services	Mothers’ responses		Fathers’ responses		Test of significance	*p*- value
	Yes	No	Yes	No		
X-ray	387 (74.4%)	408 (73.5%)	133 (25.6%)	147 (26.5%)	χ2 = 0.115	0.734
Fluoride application	361 (45.4%)	434 (54.6%)	131 (46.8%)	149 (53.2%)	χ2 = 0.158	0.691
General anesthesia	27 (93.1%)	768 (73.4%)	2 (6.9%)	278 (26.6%)	χ2 = 5.674	0.017
Root canal treatment	21 (2.6%)	774 (97.4%)	5 (1.8%)	275 (98.2%)	χ2 = 0.643	0.423
Dental scaling	304 (38.2%)	491 (61.8%)	110 (39.3%)	170 (60.7%)	χ2 = 0.096	0.757

P-value calculated by the Chi-square test. *Significant at *p* ≤ 0.05

### Distribution of dental services among study participants

**Fig. 2:**
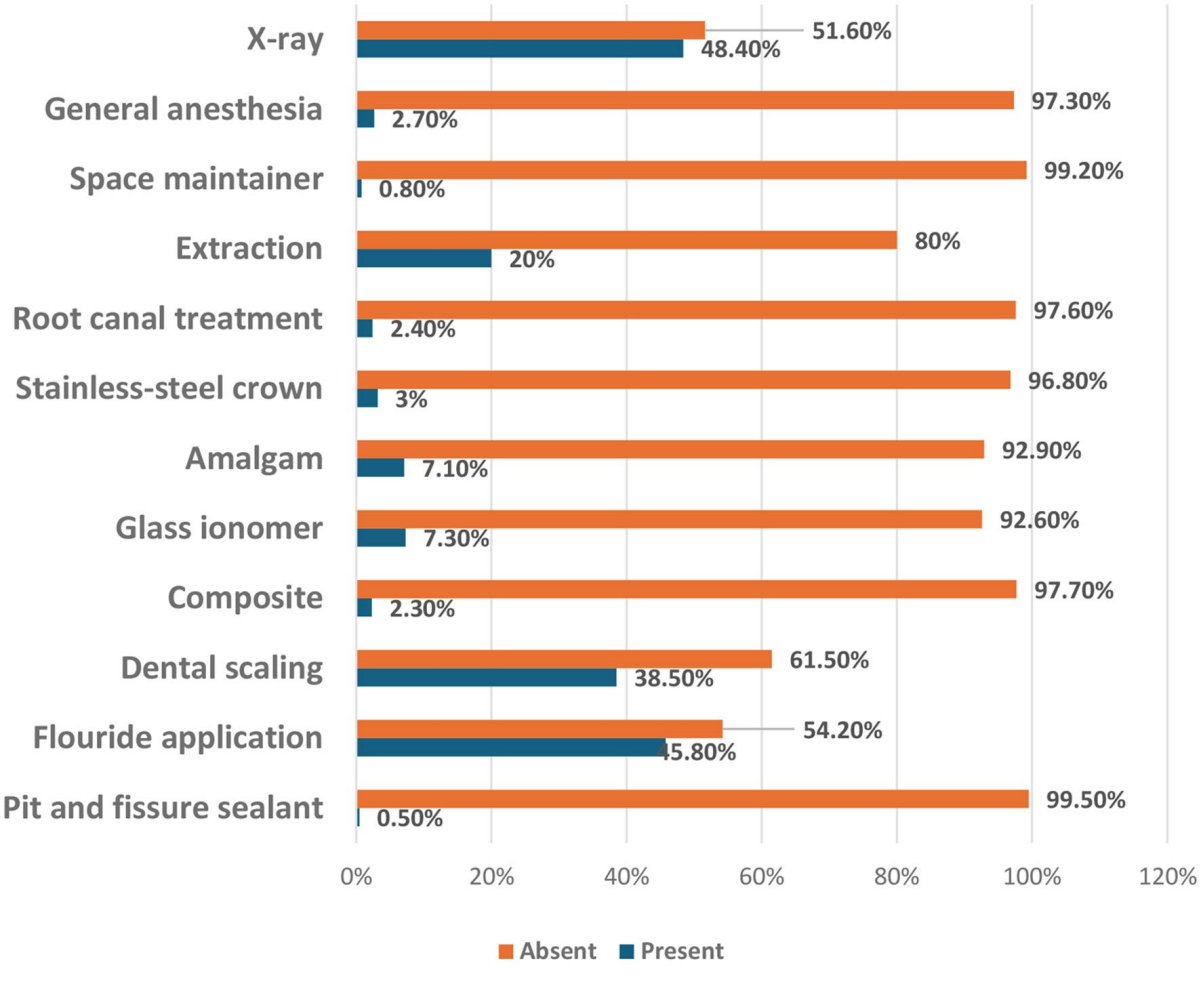
Distribution of dental service utilization among study participants

Fluoride application was the most used service (45.8%), followed by dental scaling (38.5%). Stainless steel crowns were observed in only 3% of the children, while pit and fissure sealants were the least utilized, at just 0.5%. For other dental services, extractions were the most frequently performed procedure (20%), followed by root canal treatment (2.4%) and space maintainers (0.8%). Additionally, children who performed dental X-rays or underwent general anesthesia for dental work were screened, and the results revealed that 2.7% experienced general anesthesia and 48.4% performed X-rays. (Fig. 2)

### Parents’ reported responses regarding difficult-to-detect services

As seen in Table 2, there was a statistically significant association between the utilization of dental services by children and parental reports of their children’s history of general anesthesia utilization (*p* = 0.017), with mothers being more likely to report such history (3.4%) compared to fathers (0.7%). However, no significant associations were observed between these variables regarding X-ray, fluoride application, root canal treatment, and dental scaling services.

### The impact of parental dental fear on dental caries and dental service utilization among the study participants

Dental fear was significantly correlated with dental caries in primary and permanent dentitions (deft: *r* = 0.258, *P* < 0.001), (DMFT: *r* = 0.171, *P* < 0.001). Furthermore, a significant relationship was found between the utilization of preventive restorations and parents’ dental fear (*P* < 0.001) (Tables 3 and 4).

**Table 3:** Correlation between parents’ dental fear and caries experience in permanent and primary teeth among study participants

Variables	Parents’ dental fear	
	Correlation coefficient (*r*)	*P*-value
Dental caries experience in permanent teeth (DMFT)	0.171	< 0.001*
Dental caries experience in deciduous teeth (deft)	0.258	< 0.001*

r: Spearman’s correlation. *Significant at *p* ≤ 0.05

**Table 4:** Association between parents’ dental fear and dental service utilization among study participants

Parents’ dental fear	Type of services					
	Utilized dental services	Did not utilize dental services	Total	Test of significance	*p*-value	
**Preventive restorations**						
Absent	308(33.8%)	602(66.2%)	910	χ2 = 9.560	< 0.001*	
Present	603(26.0%)	1712(74.0%)	2315			
Total	911(28.2%)	2314(71.8%)	3225			
**Conservative restorations (composite, Glass ionomer, and Amalgam)**						
Absent	48(5.3%)	862(94.7%)	910	χ2 = 9.560	0.632	
Present	132(5.7%)	2182(94.3%)	2314			
Total	180(5.6%)	3044(94.4%)	3224			
**Root canal treatment**						
Absent	11(3.1%)	344(96.9%)	355	χ2 = 1.038	0.308	
Present	15(2.1%)	705(97.9%)	720			
Total	26	1049	1075			
**Stainless steel crown**						
Absent	12(3.4%)	343(96.6%)	355	χ2 = 0.290	0.590	
Present	22(3.1%)	698(96.9%)	720			
Total	34	1041	1075			
**Space maintainer**						
Absent	4(1.1%)	351(98.9%)	355	FET	0.488	
Present	5(0.7%)	715(99.3%)	720			
Total	9(0.8%)	1066(99.2%)	1075			
**Tooth extraction**						
Absent	61(17.2%)	294(82.8%)	355	χ2 = 9.560	0.105	
Present	154(21.4%)	566(78.6%)	720			
Total	215(20.0%)	860(80.0%)	1075			
**General anesthesia**						
Absent	8(2.3%)	347(97.7%)	355	χ2 = 9.560	0.528	
Present	21(2.9%)	699(97.1%)	720			
Total	29 (2.7%)	1046(97.3%)	1075			

χ2: Chi-square test FET: Fisher’s exact test. *Significant at *p* ≤ 0.05

Preventive restoration score was estimated by adding the number of cases with pit and fissure sealants, fluoride application, and dental scaling. The conservative restoration score resulted from adding the number of cases with composite, glass ionomer, and amalgam restorations

### Association between demographic and sociodemographic variables and dental caries experiences in permanent and primary teeth

Regression analysis evaluated how demographic and sociodemographic factors affected DMFT and deft scores. Only variables with clinical relevance or statistical significance in the multivariate model were retained in the final tables. Regarding permanent teeth, the univariate analysis revealed that age (6–8 years old (*P* < 0.001), private schools (*P* = 0.038), and fathers with university education (*P* = 0.032) were negative significant predictors of DMFT scores. whereas females (*P* < 0.001) and children with birth order 3rd to 5th (*P* = 0.038) were significant positive predictors. In multivariate analysis, fathers with university education, females, and children with birth order 3rd to 5th remained significantly associated with DMFT scores in addition to Age (6–8 years old) (Table 5).

**Table 5:** Linear regression analysis of significant predictors influencing dental caries prevalence in permanent teeth among study participants

Predictors	Unadjusted (univariate) analysis				Adjusted (multivariate) analysis			
	B	95% Confidence Interval		*P*-value	B	95% Confidence Interval		*P*-value
		Lower	Upper			Lower	Upper	
**Age of children**								
6–8 years old	-0.416	-0.629	-0.204	< 0.001*	-0.322	-0.540	-0.105	0.004*
**Gender**								
Female	0.290	0.155	0.424	< 0.001*	0.248	0.111	0.385	< 0.001*
**Type of school**								
Private	-0.160	-0.306	-0.014	0.032*	-0.062	-0.233	0.107	0.470
**Number of children**								
3–5 Children	0.145	0.008	0.283	0.038*	0.152	0.009	0.294	0.047*
**Father’s education**								
University education	-0.154	-0.294	-0.013	0.032*	-0.300	-0.561	-0.039	0.023*
Model R^2^	0.052							
Model F P-value	2.692 < 0.001*							

**Dependent variable**: DMFT. *Significant at *p* ≤ 0.05

**Predictors reference categories**: Fathers’ education: less than a high school education. Number of children: 1–2 Children. Type of school: public. Gender: male. Age of children: 9–12 years old

Concerning primary teeth, univariate analysis revealed that 6-8-year-old children (*P* < 0.001), mothers aged 20–29 years (*P* = 0.028), fathers with age 30–39 years (*P* = 0.004) demonstrated a positive impact on deft scores unlike mothers & fathers with university education (*P* = 0.035 and *P* = 0.004; Respectively) and private schools were negative significant predictors. In multivariate analysis, 6-8-year children, private schools, fathers aged 30–39 years, and fathers with university education remained significantly associated with deft scores. Additionally, children with birth order 3rd to 5th or more in the family and low income (almost enough money) were significantly associated with deft scores. (Table 6)

**Table 6:** Linear regression analysis of significant predictors influencing dental caries prevalence in primary teeth among study participants

Predictors	Unadjusted (univariate) analysis				Adjusted (multivariate) analysis			
	B	95% Confidence Interval		*P*-value	B	95% Confidence Interval		*P*-value
		Lower	Upper			Lower	Upper	
**Age of the children**								
6–8 years old	1.109	0.698	1.521	< 0.001*	1.1639	0.7423	1.5855	< 0.001*
**Type of school**								
Private schools	-0.379	-0.663	-0.094	0.009*	-0.459	-0.795	-0.124	0.007*
**Number of children**								
3–5 Children	0.143	-0.125	0.410	0.269	0.281	0.013	0.575	0.040*
More than 5 children	0.734	-0.391	1.859	0.201	1.308	1.097	2.628	0.035*
**Mother’s age**								
20–29 years old	0.651	0.072	1.230	0.028*	0.276	-0.353	0.906	0.390
40–49 years old	-0.311	-0.606	-0.015	0.039*	-0.054	-0.415	0.307	0.769
**Mother’s education**								
University education	-0.272	-0.549	-0.04	0.035*	0.288	-0.298	0.875	0.335
**Father’s age**								
30–39 years old	0.400	0.131	0.668	0.004*	0.330	0.019	0.641	0.037*
**Father’s education**								
University education	-0.396	-0.668	-0.123	0.004*	-0.429	-0.827	-0.030	0.035*
**Family income**								
Almost enough money	0.029	-0.304	0.363	0.862	-0.474	-0.913	-0.35	0.034*
Model R^2^	0.064							
F P-value	3.368 < 0.001*							

**Dependent variable**: deft. *Significant at *p* ≤ 0.05

**Predictors reference categories**: Family income: borrowing money. Father’s education: high school. Father’s age: 40–49 years old. Mother’s education: less than a high school education. Mothers’ age: 30–39 years old. Number of children: 1–2 Children. Type of school: public. Age of the children: 9–12 years old

### The impact of demographic and sociodemographic variables on utilization of dental services

A logistic regression analysis evaluated how demographic and sociodemographic factors affected dental service utilization. Univariate analysis was used to determine the factors associated with dental service utilization (dependent variable) while other factors were controlled. Then, the multivariate analysis was used to determine the factors related to the dependent variable.

Univariate analysis revealed that children aged 9–12 years (*P* < 0.001) and families with substantial savings (0.006) were significantly positive predictors of dental service utilization. Conversely, male (*P* = 0.02) and attending public schools were negative predictors of dental service utilization. Then, in the multivariate logistic regression model, the same predictors showed a significant impact on the utilization of dental services, except for families with higher income. (Table 7)

**Table 7:** Binary logistic regression analysis of predictors influencing the utilization of dental services among study participants

Predictors	Unadjusted (univariate) analysis				Adjusted(multivariate) analysis			
	*P*-value	95% CI		OR	*P*-value	95% CI		OR
		Lower	Upper			Lower	Upper	
**Age of children**								
6–8 years old	Reference				Reference			
9–12 years old	< 0.001*	2.053	6.460	3.642	< 0.001*	1.634	5.344	2.955
G**ender**								
Female	Reference				Reference			
male	0.020*	0.562	0.951	0.731	0.050*	0.120	0.585	0.773
**Type of school**								
Private	Reference				Reference			
Public	< 0.001*	0.168	0.3148	0.230	< 0.001*	0.1756	0.3644	0.253
**Family income**								
Borrowed money	0.654	0.608	1.366	0.912	0.171	0.459	1.148	0.726
almost enough money	Reference				Reference			
saved money	0.006*	4.025	6.868	2.607	0.133	0.488	1.100	0.733
Model χ2 P-value					73.547 <0.001*			

**Dependent variable**: Utilization of dental services. Significant at *p* ≤ 0.05

B: Regression coefficient CI: Confidence interval OR: Odds ratio

## Discussion

This study focused on children between 6 and 12 years old during their mixed dentition phase. The school-age period is critical for a child’s growth, significantly influencing long-lasting dental health behaviors, attitudes, and beliefs [12]. The state of one’s dental health typically affects overall quality of life [7].

In this study, all children exhibited a very low care index (12.9%) and restorative index (13.1%), while the unmet treatment need index was high (86.9%). This result could be explained by the low socioeconomic level of most of the participants, as only 19.5% can save money. The study’s results align with Medina-Solis et al. [13] who noted that individuals with higher socio-economic status were more likely to receive restorative care, whereas those facing greater social disadvantages have a higher likelihood of untreated dental caries. Beyond socioeconomic factors, other reasons contribute to the low care index in dental services among Egyptian children, like; parents’ and children’s dental fear and absence of dental insurance coverage in most cases [14].

The present study revealed a low rate of dental service utilization, with fissure sealant applications being underutilized at only 0.5%. This finding is consistent with another study in Riyadh, Saudi Arabia, which observed minimal use of fissure sealants. In contrast, Veiga et al. [15] reported a much higher utilization rate of 59% for pit and fissure sealants among children. The discrepancies between these studies may be attributed to variations in economic factors, which are a significant contributor to the limited application of fissure sealants in children [16].

Limited utilization of dental services among Egyptian children can be largely attributed to systemic and policy-level factors. Oral health is not yet fully integrated into Egypt’s primary healthcare system, resulting in limited access to preventive services, especially for children in public health settings [17]. Public funding for dental health is also insufficient, leading to a scarcity of well-equipped public dental clinics, particularly in rural areas [18]. Moreover, Egypt lacks a comprehensive national oral health policy that prioritizes pediatric oral care, which contributes to uncoordinated efforts in service provision [17]. The background of dental care services in Egypt may have a possible influence on the low dental service utilization observed in our findings.

The analysis of the collected data revealed that mothers were more likely than fathers to recall and report their children having undergone general anesthesia. This tendency can be explained by the fact that mothers are frequently more actively engaged in their children’s healthcare, such as attending dental visits and engaging in discussions about treatment choices. Since general anesthesia necessitates parental consent and often involves comprehensive consultations with healthcare professionals, these responsibilities are usually assumed by mothers. Furthermore, mothers might feel a heightened concern for their child’s safety during such procedures, which could improve their memory and awareness of these occurrences. Consequently, mothers’ recollection of general anesthesia usage may be more precise and frequent compared to fathers’ [19].

Parents’ dental fear showed a significantly negative impact on the utilization of dental services such as preventive treatments. This finding aligns with Gudipaneni et al. [20], who demonstrated that elevated levels of parental dental fear result in reduced utilization of dental services, especially preventive services. This showed that parents do not seek dental treatment unless dental problems affect their quality of life [21].

Regression analysis showed that children aged 6–8 years had lower DMFT scores, higher deft scores, and lower utilization of dental services. This finding aligns with the results reported by Abbass et al. [22] They attributed the increased susceptibility of deciduous teeth to dental caries to their lower calcium content and structural differences. Additionally, caries in primary teeth may be linked to malnutrition during early childhood [23]. However, these findings aligned with Reda et al. and Clemencia [3, 24] demonstrated that younger children use dental services less frequently compared to their older counterparts. A key reason is the common parental perception that primary teeth are temporary and therefore less important, which leads to delays in seeking dental care. Moreover, primary healthcare providers may not consistently emphasize the need for early dental visits, reducing the likelihood that parents will prioritize oral health at a young age [25].

It is essential to increase parental awareness about the importance of primary teeth and implement school-based oral health screening and referral programs. Addressing transportation and accessibility barriers, especially in underserved areas, will also help bridge the gap between the need for care and actual service use.

Regarding gender, the study revealed that females had higher DMFT scores than males. The explanation typically involves three main factors: (1) girls experience tooth eruption earlier, resulting in their teeth being exposed to the cariogenic oral environment for a longer period; (2) girls have a preference for carbohydrates and sugary foods [26], which can increase their likelihood of developing dental caries; (3) physiological research has shown that women have a significantly lower average salivary flow rate compared to men, influenced by estrogen, and saliva is the medium that delivers protective agents to the oral cavity [27, 28].

Shaffer et al. [29] showed that females aged 6–11 years had better protection against dental caries than males. They attributed their results to including ethnic minorities in their sample, which may affect this difference. Additionally, Lutifyya et al. [30] illustrated a lower rate of dental service utilization among males than females. This observation aligns with the notion that society places higher importance on girls’ dental aesthetics, which explains their frequent dental visits and increased utilization of dental services.

Students enrolled in private schools exhibited lower deft, DMFT scores and higher rates of dental services utilization compared to public schools. This finding is consistent with the results reported by Mohiuddin et al. and Kumar et al. [31, 32] They explained that public school students often come from families with lower socioeconomic status and possess less knowledge about oral health, leading to poorer oral hygiene habits and reduced utilization of dental services.

Children with birth order third to fifth were significant predictors for higher DMFT and deft scores. This finding is consistent with the results reported by Sujlana and Pannu [33]. As the number of children in a family increases, parents face greater responsibilities, which can lead to less individual attention for each child. This reduced focus can negatively affect both dental and general health outcomes [34].

Mothers aged 20–29 years with children had higher deft scores compared to older mothers. This finding was aligned with Gudipaneni et al. [20] who explained that parents of older age are more knowledgeable and aware of oral health, which affects their attitudes towards children’s oral health. On the other hand, Shin et al. [35] showed that there were no significant differences between the mothers’ age and caries prevalence of their 6-11-year-old children.

Children whose parents had higher education levels showed lower DMFT and deft scores. These findings align with Abbass et al. [22] who observed an inverse significant correlation between parental education level and children’s deft. They suggested that highly educated parents focus on their children’s dental health from an early age [36]. Differences between studies can be attributed to varying age ranges; while our study focused on children up to 12 years old, others extended to 17 years. As children grow older, parental influence diminishes, potentially leading to a loss of control over the child’s dietary habits and oral hygiene practices.

According to family income, children from middle-income households, who had nearly sufficient funds, exhibited lower deft scores compared to those from low-income families who needed to borrow money. Conversely, children from high-income families, who save money, use dental services. This finding aligns with Yousaf et al. and Grytten et al. [37, 38] These relationships likely stem from low-income families facing material, social, and financial challenges that hinder their ability to care for themselves and their children. Such families encounter obstacles in accessing professional healthcare services and often fail to recognize dental issues [20, 39].

This study has several notable strengths. The relatively large sample size and inclusion of participants from diverse socioeconomic backgrounds enhance the generalizability of the findings. The use of a structured and validated questionnaire ensured consistency and reliability in data collection. Focusing on the 6–12-year age group allowed for a targeted evaluation of oral health patterns during a critical developmental stage. Additionally, combining clinical dental assessments with parent-reported data provides a more comprehensive understanding of both objective and perceived dental needs.

However, the study is subject to several limitations. Firstly, the cross-sectional nature of the study limits the ability to establish causal relationships between the observed variables and oral health outcomes. Secondly, although the study attempted to reduce recall bias by asking parents multiple questions to verify their responses, the data still relied on self-reported information that was not cross-checked with medical records. Finally, the study was conducted in a specific geographical area, which may limit the generalizability of the findings to other populations with different socio-cultural or healthcare contexts.

## Conclusion

The study concluded that children aged 6–8 years demonstrated significantly higher dental caries scores in their primary teeth. However, children aged 9–12 years showed a significantly higher rate of dental service utilization. Girls were a positive predictor of DMFT scores, while boys were a negative predictor of service utilization. Students from private schools and those whose fathers had university education demonstrated lower caries scores in both types of dentition and higher rates of dental service utilization. Children from families with more than 3 children exhibited higher caries rates in both types of dentitions. High-income families showed a positive predictor of service utilization. Dental fear was correlated with dental caries in primary and permanent dentitions. Moreover, it hindered the utilization of preventive treatments.

This study offers actionable policy recommendations to support oral health, such as implementing school-based screenings, subsidizing preventive care for low-income families, and training to reduce parental dental fear. These strategies aim to encourage early intervention and equitable access. It also calls for future longitudinal and qualitative research to explore factors like parental awareness, access to care, and school policies, to better understand age-related differences in dental service use and design more targeted interventions.

## Electronic supplementary material

Below is the link to the electronic supplementary material.

Supplementary Material 1

## Data Availability

The data supporting the findings of this study are available from the corresponding author upon reasonable request.

## Abbreviations

WHO: World Health Organization
I-CVI: The Item Validity Index
S-CVI: The Scale Validity Index
LCC: Interclass Correlation Coefficient
GA: General Anesthesia
CI: Care Index
RI: Restorative Index
UTNI: The Unmet Treatment Need Index
