# Supplementary material for: Dental caries and actual utilization of dental services among primary school children in Egypt: a cross-sectional study
Source: BMC Oral Health. 2025 Jul 15;25:1163. doi: 10.1186/s12903-025-06414-3 (PMC12265273; doi:10.1186/s12903-025-06414-3)
Supplement: Supplementary file 1 — Supplementary Material 1 [file 12903_2025_6414_MOESM1_ESM.docx]

**Supplementary file 1**

**(questionnaire)**

- **Who answers the questionnaire**
- **Mother ()**
- **Father ()**

| About the child (**demographic characteristics)** | | | | | | | | | | |
| --- | --- | --- | --- | --- | --- | --- | --- | --- | --- | --- |
| Name of the child: | | | | | | | | | | |
| *Private ()*  *Public ()* | | Type of school | |  | Name of school |  | Gender |  | Age |  |
| *Yes ()*  *No ()* | | Do child have anychronic diseas? | | | | | | | |  |

| **Age of parents** | | |
| --- | --- | --- |
| Father | | Mother |
| - *20-29 years old ()* - *30-39 years old ()* - *40-49 years old ()* - *50 years old or above ()* | - *20-29 years old ()* - *30-39 years old ()* - *40-49 years old ()* - *50 years old or above ()* | |
| **Education** | | |
| - *Less than high school ()* - *High school education ()* - *University education ()* | - *Less than high school ()* - *High school education ()* - *University education ()* | |
| **Occupation** | | |
| - Manual worker () - Prefessional jobs () - unemployed () | - Working () - Not working () | |
| **Family income** | | |
| - *Borrowed money ()* - *Almost enough money ()* - *Saved money ()* | | |

**The second part**

| **Parents’ dental fear** | | |
| --- | --- | --- |
| 1-How would you feel about the dental appointment tomorrow? | | |
| - *I expected a reasonable experience.* - *Relaxed* - *I have concerns about potential infections* - *I worry about experiencing pain* - *I'm apprehensive about the dentist's expertise* | |  |
| 2-What are your emotions while waiting in the dentist's reception area? | | |
| - *Relaxed ()* - *somewhat fearful ()* - *fearful ()* |  | |
| 3- How do you feel while you are sitting in the dental chair as the dentist prepares the instruments? | | |
| - *Relaxed ()* - *Somewhat fearful ()* - *Fearful ()* | | |
| 4-What do you feel when the dentist works near your gum and teeth? | | |
| - *relaxed ()* - *somewhat fearful ()* - *fearful ()* | |  |
